# Willingness toward kidney donation among patients’ relatives at Muhimbili National Hospital, Dar es Salaam, Tanzania: A cross-sectional study

**DOI:** 10.1371/journal.pone.0351952

**Published:** 2026-07-10

**Authors:** Maua Nyagawa, Baraka Morris, Suleiman Chombo, Francis Furia

**Affiliations:** 1 Department of Clinical Nursing; Nephrology Nursing, School of Nursing, Muhimbili University of Health and Allied Sciences (MUHAS), Dar es Salaam, Tanzania; 2 Department of Nursing Management, School of Nursing, Muhimbili University of Health and Allied Sciences (MUHAS), Dar es Salaam, Tanzania; 3 Department of Epidemiology and Biostatistics, School of Public Health and Social Sciences, Muhimbili University of Health and Allied Science, Dar es Salaam, Tanzania; 4 Department of Pediatrics and Child Health, School of Medicine, Muhimbili University of Health and Allied Sciences (MUHAS), Dar es Salaam, Tanzania; National Research Centre, EGYPT

## Abstract

**Background:**

Kidney transplantation provides superior long-term survival and quality of life over dialysis for patients with end-stage kidney disease; however, its use is limited by the availability of donors. In Tanzania, only living-related kidney transplantation has been performed since 2017 at Muhimbili National Hospital (MNH). Success relies on donor readiness, yet little is known about public willingness to donate.

**Aim:**

To assess willingness of patients’ relatives to donate kidneys at MNH.

**Methods:**

Cross-sectional study design among 424 in-patient relatives at MNH from May to June 2023. Systematic random sampling was used to recruit participants. Data were collected using a questionnaire comprised of inquiries on socio-demographics, knowledge, attitudes, and willingness to donate and analyzed using the Stata 18 software. Frequency distribution tables summarized descriptive statistics. Modified Poisson regression with robust variance identified factors associated with willingness.

**Results:**

Of the 424 participants, Mean age 36 ± 11 years; 240(56.6%) female, 362(85.4%) urban, 400(94.3%) educated. While 361(85%) heard of organ donation, only 32(7.5%) had adequate knowledge, 289(68.2%) positive attitude, 200(47.2%) willing to donate. Age 35–44 years (aRR = 0.69 [95% CI: 0.49–0.99], p = 0.046), female gender (aRR = 0.82 [95% CI: 0.67–0.99], p = 0.042), informal traders/farmers (aRR = 0.77 [95% CI: 0.60–0.99], p = 0.041) had lower willingness versus counterparts. Positive attitude showed 74% higher likelihood (aRR = 1.74 [95% CI: 1.31–2.30], p < 0.001).

**Conclusion:**

Low kidney donation willingness was influenced by attitude, age, gender, and occupation. Majority of the participants lacked adequate knowledge. Educational programs needed to improve knowledge, attitude, and willingness.

## Introduction

Organ transplantation is a life-saving treatment involving the surgical replacement of failing organs using donated healthy ones. The success of a transplantation program depends on the sustainable availability of organs [1,2]. Unfortunately, there is a global scarcity of donor organs, while the demand is increasing. Organs are available for less than 10% of all needy individuals, translating into an organ shortage crisis [1]. In the United States, the kidney transplantation waiting list grew by 18% between 2020 and 2022, leading to longer waiting times and increased patient morbidity and mortality [2]. Sub-Saharan Africa (SSA) bears the world’s highest CKD burden (prevalence 10–20% stages 1–5), with end stage renal disease (ESRD) incidence 100–200 pmp but prevalence <50 pmp due to treatment gaps [3,4]. Likewise, SSA is the region with the lowest availability of transplant services for the treatment of CKD, which results in high mortality [3,4]. Dialysis mortality reaches 31% in SSA (17% Tanzania), driven by inadequate hemodialysis (HD) quality [5,6]. Annual dialysis costs average $18,741 (South Africa $39,102; Tanzania $37,261) equals to>20x GDP/capita for most countries, causing catastrophic expenditures where insurance covers only <8% of the population [7,8]. By 2030, > 70% global ESRD patients will be African [8]. Tanzania exemplifies this crisis with CKD prevalence 7–15% general population, and 83.7% hospitalizations [9]. Currently the country has 3231 patients on dialysis across 47 HD units with 649 machines [10].

Over the past three decades, countries like South Africa, Kenya, Ghana, Nigeria, Uganda, and Sudan have established kidney transplantation [5]. Most of these countries rely on living donation transplantation, except South Africa, which uses both living and deceased donors.

Tanzania began offering kidney transplantation in 2017, with two hospitals currently providing this critical service. Similar to many other countries in the region, Tanzania offers living-related kidney transplantation [6]. Since then, only 88 living kidney transplants done at MNH [11]. Major barriers includes cost, donor shortage (living-related donors only), nephrologist shortage (31 super specialists) [7,8]. Sustaining this program requires effective donor mobilization, which relies heavily on strong community engagement and support. Raising community awareness is essential for encouraging potential donors to participate. Increased awareness can help dispel negative attitudes, misconceptions, and cultural concerns that often deter organ donation. Furthermore, it promotes a better understanding of living-related kidney transplants, alleviates fears, and highlights the benefits of organ donation factors that are crucial for the sustainability and success of Tanzania’s kidney transplantation program [7–10].

Effective community engagement requires understanding existing knowledge, attitudes, and willingness toward kidney transplantation among potential donors. However, limited data exists on kidney transplant knowledge or donation willingness among patient relatives in Tanzania. This study therefore assessed knowledge, attitudes, and willingness to donate kidneys among in-patient relatives at Muhimbili National Hospital (MNH) to inform targeted donor mobilization strategies.

## Materials and methods

### Ethics process

This study was approved by the Muhimbili University of Health and Allied Sciences (MUHAS) Institutional Review Board (IRB) with reference number MUHAS-REC-04-2023-1631. MNH administration granted permission to conduct this study in the hospital. Each participant was requested to provide a written informed consent before recruitment. Access to the information collected was granted strictly to persons involved in this study.

### Study setting

This cross-sectional study was conducted among in-patient relatives at Muhimbili National Hospital (MNH) in Dar es Salaam, Tanzania, from May 4 to June 4, 2023. MNH is a national referral hospital with bed capacity of 2178 (1570 beds at main Upanga campus and 608 beds at Mloganzila facility, 23 km away) [12]. MNH serves 2,000−3,000 outpatients and 1,500−2,000 inpatients daily. It provides chronic hemodialysis for approximately 400 patients. Peritoneal dialysis is not routinely available; it is predominantly used for acute cases, mostly in children. The hospital operates as a public government national referral hospital. It serves patients with insurance, self-paying patients, and those who have some or all costs waived. MNH is one of the two hospitals in Tanzania that offer kidney transplantation services. Since 2017, a total of 88 living related kidney transplant surgeries have been conducted at MNH. In Tanzania, only living-related kidney donations are currently practiced in both hospitals under the MNH renal services regulations of the year 2017. The regulations specifically define ‘near relatives’ and prohibit unrelated or distant relative donations. This restriction limits the donor pool but ensures ethical oversight in Tanzania’s emerging transplant program. However, the country lacks comprehensive national legislation governing organ or tissue donation and transplantation. Deceased organ donation and tissue donation (such as corneas or bones) are not yet performed in Tanzania.

### Study population

Study participants included all in-patient relatives who visited their hospitalized family members in five inpatient blocks, including surgical, medical, maternity, and paediatrics at MNH.

### Study variables

#### Dependent variable.

The outcome variable for this study was willingness to donate kidneys coded as 1 “if the participant was willing to donate” and 0 “if the participant was not willing”.

#### Independent variables.

The regression model included socio-demographic variables as independent predictors: gender (male vs. female), age categorized as 18–24 years, 25–34 years, 35–44years and >45years, place of residence (rural vs. urban), educational attainment (no formal education, primary, secondary, and college/degree or higher), occupation (formally employed, informal employment/traders, and dependents), marital status (married/cohabiting vs. single/widowed/divorced), and awareness of kidney donation (ever heard vs. never heard). All variables were entered as categorical factors using appropriate reference categories. Other covariates included knowledge of kidney donation and attitudes towards organ donation. These variables were selected based on prior studies showing associations with organ donation in SSA contexts [13].

### Sampling and recruitment process

**Fig 1:**
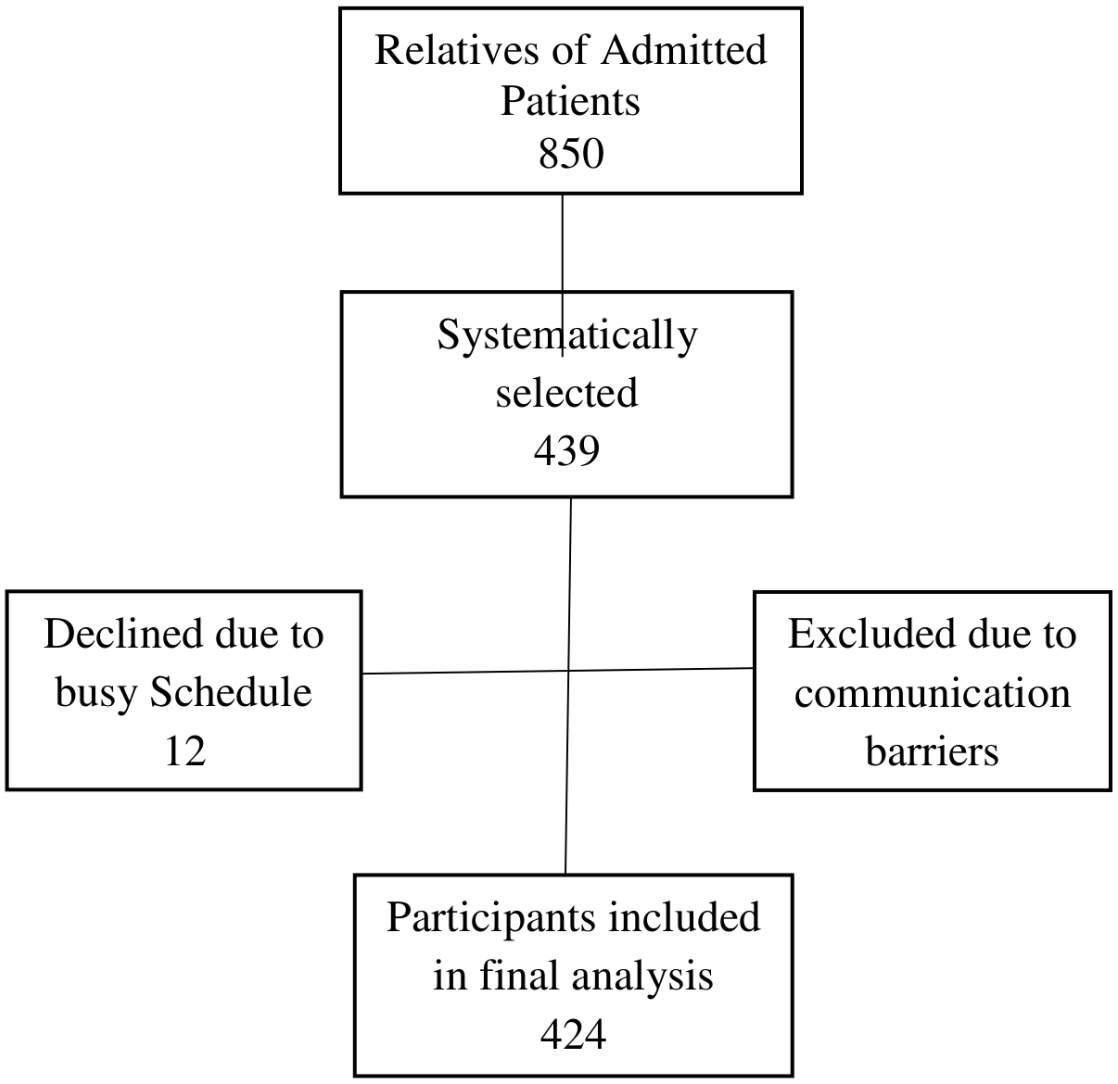
Study participant recruitment flowchart. A systematic random sampling method was used to recruit participants. From a list of 850 admitted patients during the study period, the sampling interval was calculated by dividing the patient list by the target sample size (850/424 ≈ 2). The first participant was selected randomly, followed by the recruitment of every second patient’s relative (k = 2). For patients with multiple eligible relatives, one was selected via simple random sampling.

The sample size for this study was calculated using Cochran’s formula for infinite populations, with a willingness prevalence of 54% based on the study done in Uganda [13], a 95% confidence interval, and a 5% margin of error. After adjusting for a 10% non-response rate, the minimum required sample size was 424. Of 439 eligible participants approached, 15 were excluded due to refusal or communication barriers, resulting in 424 participants for analysis (Fig 1).

### Data collection

A structured, self-administered questionnaire was adapted from a tool used in a 2018 study by Singh et al. in Qatar (knowledge and attitude Cronbach’s alpha were 0.90 and 0.67 respectively) [14]. The tool was originally in English and was later translated into Kiswahili. Experts from MNH nephrology team reviewed the questionnaire to confirm Tanzania cultural relevance. The questionnaire was piloted among 40 in-patient relatives at MNH (excluded from main study). Pilot identified frequent ‘I don’t know’ responses to knowledge questions; I don’t know’ option added to improve response accuracy. The Cronbach’s alpha coefficients for the knowledge and attitude sections were 0.70 and 0.72, respectively. Confirmed acceptable reliability.

The final questionnaire consisted of four sections to collect information on participants’ socio-demographic characteristics, knowledge of organ donation, attitudes toward donation, and willingness to donate organs. The data were collected in a private areas or waiting rooms of the wards. The survey was answered by using paper and pen as both self-administered questionnaires for literate participants and researcher administered face-to-face interviews for those unable to read/write. Principal researcher and 3 trained assistant researchers were available to provide assistance and clarifications upon request.

Willingness to donate was determined by a simple “yes” or “no” response**.** Knowledge of kidney donation was assessed using a composite score based on 22 questions, with each correct response scored as 1 and each incorrect response as 0. The total score ranged from 0 to 22, participant who scores 12–22 points, was coded as 1 “having adequate knowledge”, otherwise, was coded as 0 ‘inadequate knowledge. A participant was considered as having adequate knowledge if the participant scored above average (above 50 percentile). This cut-off was chosen based on similar studies to reflect a moderate level of understanding [15,16]. Attitudes were assessed using 13 items on a five-point Likert scale (strongly agree to strongly disagree). Responses were initially coded from 1 (strongly agree) to 5 (strongly disagree). For positively phrased items, scores were reverse-coded so that higher values reflected more favorable attitudes. Negatively phrased items were kept in the original direction. For each item 1–3 scores was coded as 0 and 4–5 scores was coded as 1. The total score was then summed. Participant who scored 7–13 points was coded as 1 “positive attitude”, otherwise was coded as 0 “negative attitude”. Higher standardized scores indicate a more positive attitude. Scores above the mean were considered a positive attitude*.* Socio-demographic characteristics were assessed using 6 questions were participants reported their; age, gender, residence, education level, occupation, and marital status.

### Data analysis

Data were entered, cleaned, and analyzed using the Stata 18 software. Both descriptive and inferential statistical methods were applied. Descriptive statistics were summarized using frequency distribution tables. Factors associated with willingness to donate were examined using both univariable and multivariable modified Poisson regression models with robust variance estimators. Variables were considered for inclusion in the multivariable model based on a p-value threshold of <0.20 in univariable analysis, as well as prior evidence from the literature and substantive knowledge regarding potential confounders. The modified Poisson regression approach with robust variance estimation was used to directly estimate prevalence ratios, as the outcome of interest was common (prevalence >10%). Logistic regression was not preferred because odds ratios may overestimate the association when outcomes are common [17–19]. Statistical significance was assessed at a p-value of ≤ 0.05. Chi-square test of association was also performed to determine the nature of association between each of the examined covariates and the willingness to donate. The significance was also determined at p ≤ 0.05.

## Results

### Study participants’ characteristics

**Fig 2:**
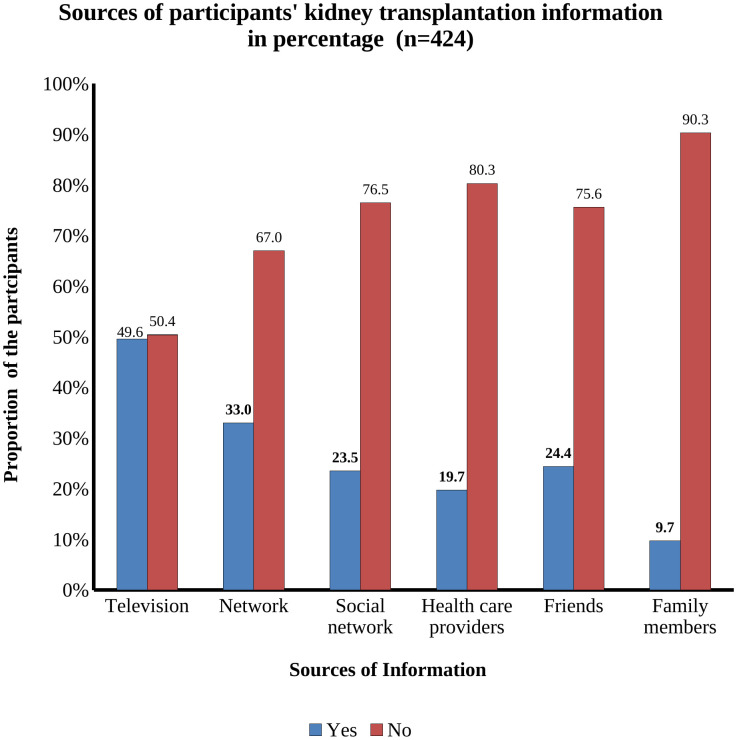
Source of participants’ kidney transplantation information.

Of the 424 study participants, 240(56.6%) were female. Participants aged 25–34 years constituted the largest proportion 148 (34.9%), followed by those aged 35–44 years 106(25%), while 96(23%)were aged 45 years and above. The majority of participants were married or cohabiting 277(65.3%) and informal traders or subsistence farmers 230(54%). Three hundred sixty one (85%) participants had heard of kidney donation. The predominant source of information was television 179(49.6%), primarily referring to Tanzanian local channels broadcast in Swahili, followed by the internet 119(33%), friends 88(24.4%), and social media networks 85(23.5%) (Fig 2).

Only 32(7.5%) of participants demonstrated adequate knowledge of kidney donation, whereas 289(68%) expressed a positive attitude toward organ donation. Overall, 200(47%) of the participants reported being willing to donate kidneys. Among those willing to donate (n = 200), 117 (58.5%) chose living donation, 45 (22.5%) chose cadaveric donation, 38 (19%) indicated willingness to do both and 174 (87%) indicated willingness to consent to organ donation for their loved ones after death (see Table 1).

**Table 1:** Respondent characteristics (n = 424).

Variable	Frequency	Percent
**Mean age (SD)**	**36 (11)**	
**Median age (IQR)**	**34(27,43)**	
**Age**		
18-24	74	17.5
25-34	148	34.9
35-44	106	25.0
45+	96	22.6
		
**Sex**		
Male	184	43.4
Female	240	56.6
		
**Permanent residence**		
Rural	62	14.6
Urban	362	85.4
		
**Education**		
Non formal	24	5.7
Primary	102	24.1
Secondary	149	35.1
College/Degree	149	35.1
		
**Occupation**		
Formally employed	81	19.1
Informal traders/subsistence agriculture	230	54.2
Dependent	113	26.7
		
**Marital status**		
Married/cohabiting	277	65.3
Single/divorced/widow	147	34.7
		
**Awareness on organ donation (ever heard of kidney donation)**		
Yes	361	85.1
No	63	14.9
		
**Mean knowledge score** (SD)	4.9 (2.3)	
**Median knowledge score** (IQR)	5 (3,6)	
		
**Mean attitude score** (SD)	5.2 (2.7)	
**Median attitude score** (IQR)	5 (3,7)	
		
**Knowledge of organ donation**		
poor knowledge	392	92.5
adequate knowledge	32	7.5
		
**Attitude towards organ donation**		
negative attitude	135	31.8
positive attitude	289	68.2
		
**Willingness to donate**		
Willing	200	47.2
Not willing	224	52.8
		
**Willing to living donation only (n = 200)**		
Yes	117	58.5
No	83	41.5
		
**Willing to cadaveric donation only (n = 200)**		
Yes	45	22.5
No	155	77.5
		
**Willing to both living and cadaveric donation (n = 200)**		
Yes	38	19
No	162	81
		
**Willing to consent for beloved ones’ donation after their death (n = 200)**		
Yes	174	87
No	26	13

**SD: standard deviation; IQR: interquartile range**

### Factors associated with willingness to donate

The results from Table 2 on the multivariable modified Poisson regression model with robust estimator indicated that participants aged 35–44 years had 31% less likelihood (aRR = 0.69, [95% CI: 0.49–0.99], p < 0.046), of willingess to donate compared to those aged 18–24 years. Female participants indicated 18% less likelihood (aRR = 0.82, [95% CI: 0.67–0.99], p < 0.042) of willingness to organ donation compared to their male counterparts. Participants who are informal traders/subsistence farmers had 23% (aRR = 0.77, [95% CI: 0.60–0.99], p < 0.041) less likelihood of willingness to kidney donation compared to their counterparts who are formally employed. A positive attitude towards organ donation indicated 74% (aRR = 1.74, [95% CI: 1.31–2.30], p < 0.001) higher likelihood of willingness to donate compared to their counterparts with negative attitude. Though, it was not found to be significant at 95% confidence level, but participants with adequate knowledge indicated 26% [95% CI:0.95–1.67] higher likelihood of willingness to organ donation compared to their counterparts with inadequate knowledge on organ donation.

**Table 2:** Factors associated with willingness to donate (n = 424).

Variable	Willingness to donate		Univariable model		Multivariable model	
	Willing category, N (%)	Chi2 p-value	crudeRR[95% CI]	p-Value	adjustedRR[95% CI]	p-Value
**Age**						
18-24	40(54.1)	0.307	Ref		Ref	
25-34	73(49.3)		0.91 [0.70-1.19]	0.500	0.76[0.57-1.03]	0.075
35-44	43(40.6)		0.75 [0.55-1.03]	0.072**	0.69 [0.49-0.99]	0.046**
45+	44(45.8)		0.85 [0.63-1.15]	0.285	0.75 [0.53-1.06]	0.104
**Sex**						
Male	100(54.3)	0.010**	Ref	.	Ref	
Female	100(41.7)		0.77 [0.63-0.94]	0.009**	0.82 [0.67-0.99]	0.042**
**Permanent residence**						
Rural	22(35.5)	0.046**	Ref		Ref	
Urban	178(49.2)		1.39 [0.97-1.97]	0.069	1.27 [0.89-1.83]	0.192
**Education**						
Non formal	7(29.2)	0.063	Ref		Ref	
Primary	43(42.2)		1.45 [0.74-2.81]	0.277	1.33 [0.69-2.55]	0.398
Secondary	69(46.3)		1.59 [0.83-3.03]	0.162	1.20 [0.63-2.29]	0.573
College/Degree	81(54.4)		1.86 [0.98-3.54]	0.057	1.22 [0.64-2.33]	0.547
**Occupation**						
Formally employed	48(59.3)	0.053	Ref		Ref	
Informal traders/subsistence agriculture	102(44.3)		0.75 [0.59-0.94]	0.014**	0.77 [0.60-0.99]	0.041**
Dependent	50(44.2)		0.75 [0.57-0.98]	0.037**	0.76 [0.56-1.05]	0.095
**Marital status**						
Married/cohabiting	130(46.9)	0.893	Ref		Ref	
Single/divorced/widow	70(47.6)		1.01 [0.82-1.25]	0.893	0.96 [0.75-1.22]	0.714
**Knowledge**						
poor knowledge	180(45.9)	0.071	Ref		Ref	
adequate knowledge	20(62.5)		1.36 [1.02-1.82]	0.037**	1.26 [0.95-1.67]	0.111
**Attitude**						
negative attitude	40(29.6)	<0.001**	Ref		Ref	
positive attitude	160(55.4)		1.87 [1.41-2.47]	<0.001**	1.74 [1.31-2.30]	<0.001**

**◊indicates significance at 95% confidence level, p ≤ 0.05 was considered statistically significant

## Discussion

This study found that knowledge of kidney donation was low, despite a majority demonstrating positive attitudes and nearly half expressing willingness to donate. Attitudes, occupations, age, and sex influenced participants’ willingness. The strikingly low knowledge level (7.5%) contrasts with higher rates found among adults in Saudi Arabia (48.4%) and university students in Oman (34.1%) [16,20]. Healthcare workers in Iran showed even greater awareness (nurses 62.8%, physicians 66.7%) [21]. This disparity likely reflects differences in education, professional exposure, and cultural contexts. The lower knowledge of kidney donation in this study aligns with sub-Saharan Africa (SSA) patterns, where limited public awareness, inadequate education programs, cultural misconceptions, and religious influences hinder comprehensive understanding despite rising transplant needs [22]. Notably, over 50% positive attitudes despite low knowledge reflect a recognized SSA paradox, where humanitarian values like compassion and moral/religious obligations outweigh informational gaps [23]. These findings highlight the critical need for public education campaigns to improve kidney donation knowledge in Tanzania, as community awareness directly impacts donor mobilization.

Most participants were aware of kidney donation services in Tanzania but were unfamiliar with the concept of donation after death (brain or cardiopulmonary death). Limited awareness of deceased donation stems from how organ donation is portrayed in the country. Information sources such as television, radio, online media, social media, peers, and healthcare providers have primarily emphasized living-related transplants. Living-related kidney and bone marrow transplantation have been practiced in Tanzania for the past seven years [18,19] shaping public narratives toward these programs. Similar patterns of awareness have been reported in Uganda, Ethiopia, and Nigeria [13,24–26]. These findings highlight both the media’s role in raising awareness and the need to expand donation initiatives beyond living donors.

Attitude towards donation play an important role in sustaining organ donation efforts. Both Positive and negative attitudes have been documented in several reports. Abiodun et al. reported negative attitudes toward organ donation among healthcare workers in Nigeria. Similar findings were observed by Mpekethu et al. in a study conducted among college students in Kenya [25,27]. More than 50% of participants in our study had a positive attitude toward donation, which is an interesting phenomenon that highlights the potential for a successful kidney donation program in Tanzania. Several factors may influence society’s attitude towards various health initiatives, such as organ donation, including community awareness, social support structure, and cultural values.

Participants with positive attitudes toward organ donation were nearly twice willing to donate compared to those with negative attitudes. Similar to other African studies, overall willingness to donate was low [13,24,28,29]. Among those willing, 58.5% preferred living donation, 22.5% cadaveric donation, and 19% both. A notable disparity emerged regarding posthumous donation—while 87% supported deceased donation for loved ones, only 22% consented for themselves, suggesting cultural or emotional influences. Positive public attitudes have been shown to increase willingness to donate [13,24,30], yet persistent fears regarding the donation process, body manipulation, and healthcare system distrust sustain negative attitudes [31]. These findings collectively highlight how cultural norms, emotional factors, distrust in medical systems, and public knowledge fundamentally drive attitude formation toward kidney donations.

Tanzania’s ongoing efforts to amend hospital regulations and acknowledge the importance of organ transplantation indicate that with sustained political will, capacity building, and ethical governance, deceased donation programs can be developed to address critical organ shortages and improve patient outcomes. The efforts must also consider traditional African religious and cultural norms often emphasizing the spiritual importance of bodily integrity after death [32,33]. These beliefs can be eliminated through culturally sensitive education and community dialogues to build trust and acceptance.

The study found that participants with formal employment were significantly more willing to donate kidneys compared to those engaged in informal trading or subsistence agriculture. This may be attributed to greater exposure to accurate health information and a better understanding of the benefits and risks of organ donation. This association aligns with findings from Tanzania and other low- and middle-income countries, where education and employment status are key determinants of health awareness, decision-making capacity, and participation in preventive health behaviors [34–36]. The lower willingness among informal traders and subsistence agriculture workers to donate kidneys is multi-factorial; limited health literacy, strong cultural-religious beliefs about bodily integrity after death, mistrust rooted in socio-economic realities, and economic hardship all contribute collectively to this phenomenon [32,37].

Willingness to donate was also influenced by socio-demographic characteristics where participants aged 35–44 years were less willing to donate compared to those aged 18–24 years [38]. This may be due to the fact that mid-adulthood brings heightened life responsibilities and increased health concerns compared to younger adults who often lack dependents and display higher altruism driven by optimism and less exposure to aging-related fears [39]. Female participants were less likely to donate compared to male participants. This may be due to African culture that does not give much freedom to women to make decisions on their own [40]. On contrary to the study done in Uganda, female were more willing to donate compared to male [13]. This could have attributed by the fact that women are more empathetic than male [41]. Gender variations in kidney donation willingness are deeply rooted in cultural norms that limit women’s decision-making autonomy and religious beliefs, as noted by Akinyemi et al. who found education and gender shaped organ donation intent among older Nigerians and Yee et al. who highlighted sex disparities in equitable donor pools stemming from societal factors [40,41].

### Study strengths and limitations

The study documents on kidney donation willingness among relatives in Tanzania, addressing critical evidence gap in sub-Saharan Africa where transplant services are emerging. A strong sample size of 424 participants enhances statistical power for detecting socio-demographic associations. Use of a validated, adapted questionnaire ensures reliability, with Cronbach’s alpha values of 0.70–0.72 confirming internal consistency across knowledge, attitude, and willingness domains. Employment of modified Poisson regression for adjusted risk ratios (aRR) appropriately handles the common outcome prevalence (47.2%), providing accurate effect estimates. Findings offer direct policy relevance for donor mobilization in Tanzania’s growing kidney transplant program at Muhimbili National Hospital, highlighting low knowledge (7.5%) yet positive attitudes (>50%) to guide targeted education campaigns. As the first such study in the region targeting relatives (a high-potential donor pool) it establishes a baseline for future interventions amid rising CKD burdens.

Our study has some limitations, including the single setting context that limits the generalizability of the findings. The cross-sectional design does not provide for the establishment of causal relationships between variables and outcomes. The use of binary format, although it reflects real-life decisions, we acknowledge that a Likert scale could have offered more insights into willingness levels. Also consenting to a deceased family member’s donation, was assessed only to 200 participants who expressed personal willingness to donate, this may overestimated the proportion willing to consent as it excluded those unwilling to donate personally who might still consent for a relative. Furthermore, the study methodology did not assess whether those surveyed family members (only one per family) would be able to donate based on comorbidities such as high Body Mass Index **(**BMI). The study could not evaluate variations within family support groups for patients.

## Conclusion

The study revealed that about half of the participants expressed a willingness to donate their kidneys. Participants with a positive attitude toward organ donation were more likely to be willing to donate. Female participants, middle adults, and informal traders/subsistence farmers showed less willingness towards kidney donation than their counterparts. Media campaigns targeting low-education groups and informal employment sectors (traders, subsistence farmers) and community in general are needed to boost Tanzania’s living kidney donor pool.

## Supporting information

S1 Fig Study participant recruitment flowchart. (DOCX)

S2 Fig Source of participants’ kidney transplantation information. (DOCX)

S3 File Ethical clearance. (DOCX)

S4 File Data set. (XLSX)

S5 File Atitude table. (DOCX)

S6 File Knowledge table. (DOCX)

S7 File Questionnaire. (DOCX)

S8 File Normality results. (DOCX)

S9 File Strobe. (DOC)

S10 File Clinical study checklist. (DOCX)
