# Supplementary material for: Willingness toward kidney donation among patients’ relatives at Muhimbili National Hospital, Dar es Salaam, Tanzania: A cross-sectional study
Source: PLoS One. 2026 Jul 10;21(7):e0351952. doi: 10.1371/journal.pone.0351952 (PMC13353935; doi:10.1371/journal.pone.0351952)
Supplement: S1 Fig — (DOCX) [file pone.0351952.s001.docx]

***Figure 1:*** ***Study participant recruitment flowchart***.

Relatives of Admitted Patients

850

Systematically selected

439

Excluded due to communication barriers

3

Declined due to busy Schedule

12

Participants included in final analysis

424
