# Supplementary material for: Willingness toward kidney donation among patients’ relatives at Muhimbili National Hospital, Dar es Salaam, Tanzania: A cross-sectional study
Source: PLoS One. 2026 Jul 10;21(7):e0351952. doi: 10.1371/journal.pone.0351952 (PMC13353935; doi:10.1371/journal.pone.0351952)
Supplement: S2 Fig — (DOCX) [file pone.0351952.s002.docx]

***Figure 2: Source of participants’ kidney transplantation information***
