# Supplementary material for: Willingness toward kidney donation among patients’ relatives at Muhimbili National Hospital, Dar es Salaam, Tanzania: A cross-sectional study
Source: PLoS One. 2026 Jul 10;21(7):e0351952. doi: 10.1371/journal.pone.0351952 (PMC13353935; doi:10.1371/journal.pone.0351952)
Supplement: S5 File — (DOCX) [file pone.0351952.s005.docx]

| Item | Response, N (%) | | | | |
| --- | --- | --- | --- | --- | --- |
|  | **Strongly Agree** | **Agree** | **Neutral** | **Disagree** | **Strongly disagree** |
| 1. Organ donation is a good thing and should be promoted | 46(10.8) | 79(18.6) | 3(0.7) | 155(36.6) | 141(33.3) |
| 1. Organ donation saves somebody’s life | 23(5.4) | 31(7.3) | 2(0.5) | 133(31.4) | 235(55.4) |
| 1. Donating will impact my life after death in a good way | 81(19.1) | 119(28.1) | 1(0.2) | 116(27.4) | 107(25.2) |
| 1. Organ donation will be rewarded by God | 59(13.9) | 79(18.6) | 6(1.4) | 115(27.1) | 165(38.9) |
| 1. My family will not allow donation of my organs | 94(22.2) | 145(34.2) | 9(2.1) | 86(20.3) | 90(21.2) |
| 1. Organ donation might leave me weak and disabled | 65(15.3) | 106(25.0) | 3(0.7) | 141(33.3) | 109(25.7) |
| 1. I don’t trust the health care system and it is better to go abroad for organ donation and transplantation | 121(28.5) | 150(35.4) | 1(0.2) | 66(15.6) | 86(20.3) |
| 1. I am not healthy to donate | 84(19.8) | 138(32.5) | 6(1.4) | 98(23.1) | 98(23.1) |
| 1. My age is not fit for organ donation | 118(27.8) | 149(35.1) | 4(0.9) | 70(16.5) | 83(19.6) |
| 1. The operation procedure for procuring organs is discouraging | 90(21.2) | 129(30.4) | 2(0.5) | 94(22.2) | 109(25.7) |
| 1. Organ retrieval process after death may cause body disfigurement | 141(33.3) | 122(28.8) | 3(0.7) | 80(18.9) | 78(18.4) |
| 1. In case of an emergency; doctors will not provide enough care if the patient is a known organ donor | 121(28.5) | 143(33.7) | 3(0.7) | 86(20.3) | 71(16.7) |
| 1. I believe in the burial of an intact body | 88(20.8) | 94(22.2) | 1(0.2) | 103(24.3) | 138(32.5) |

Table 3: Participants' responses regarding their attitude toward organ donation
