# Supplementary material for: Willingness toward kidney donation among patients’ relatives at Muhimbili National Hospital, Dar es Salaam, Tanzania: A cross-sectional study
Source: PLoS One. 2026 Jul 10;21(7):e0351952. doi: 10.1371/journal.pone.0351952 (PMC13353935; doi:10.1371/journal.pone.0351952)
Supplement: S6 File — (DOCX) [file pone.0351952.s006.docx]

| Item | Response, N (%) | | |
| --- | --- | --- | --- |
| 1. Kidney donation means; a donation | a. From living donor to the patient 283(66.7)  b. From dead donor to the patient 15(3.5)  c. All of the above 56(13.2)  d. I do not know 70(16.5) | | |
| 1. Age at which an individual can donate a kidney | a. Above 18 years 289(68.2)  b. Any age 18(4.2)  c. I don’t know 117(27.6) | | |
|  | **Yes** | **No** | **I don’t know** |
| 1. Diabetes and Hypertension are the most common causes of CKD | 123(29.0) | 100(23.6) | 201(47.4) |
| 1. Is possible to donate all my two kidneys after death | 115(27.1) | 123(29.0) | 186(43.9) |
| 1. Is it feasible for someone who is brain dead to recover from their injuries | 30(7.1) | 189(44.6) | 205(48.3) |
| 1. Donating kidney is safe | 223(52.6) | 63(14.9) | 138(32.5) |
| 1. Other organs/tissues that can be donated |  |  |  |
| - 1. Heart | 68(16.0) | 299(70.5) | 57(13.4) |
| - 1. Liver | 54(12.7) | 313(73.8) | 57(13.4) |
| - 1. Lungs | 27(6.4) | 340(80.2) | 57(13.4) |
| - 1. Pancreases | 19(4.5) | 348(82.1) | 57(13.4) |
| - 1. Intestine | 18(4.2) | 349(82.3) | 57(13.4) |
| - 1. Blood | 291(68.6) | 76(17.9) | 57(13.4) |
| - 1. Cornea | 31(7.3) | 336(79.2) | 57(13.4) |
| - 1. Skin | 35(8.3) | 332(78.3) | 57(13.4) |
| - 1. Bone marrow | 55(13.0) | 312(73.6) | 57(13.4) |
| - 1. Bone | 19(4.5) | 348(82.1) | 57(13.4) |
| 1. Is it possible to donate one of my two kidneys during my life | 302(71.2) | 65(15.3) | 57(13.4) |
| 1. My religion allows organ donation | 240(56.6) | 34(8.0) | 150(35.4) |
| 1. I know someone who has donated an organ | 336(79.2) | 88(20.8) |  |
| 1. I cared for a patient with a chronic disease | 188(44.4) | 235(55.6) |  |
| 1. I cared for a relative with kidney disease | 95(22.4) | 329(77.6) |  |

Table 2: Participants' responses regarding to their knowledge on organ donation
