# Supplementary material for: Willingness toward kidney donation among patients’ relatives at Muhimbili National Hospital, Dar es Salaam, Tanzania: A cross-sectional study
Source: PLoS One. 2026 Jul 10;21(7):e0351952. doi: 10.1371/journal.pone.0351952 (PMC13353935; doi:10.1371/journal.pone.0351952)
Supplement: S7 File — (DOCX) [file pone.0351952.s007.docx]

# APPENDICES

## Appendix I Questionnaires (English Version)

ID NO…… DATE….../….../2023

**TITTLE:** WILLINGNESS TOWARDS ORGAN DONATION AMONG PATIENTS’ RELATIVES AT MUHIMBILI NATIONAL HOSPITAL.

**PART I. SOCIODEMOGRAPHIC CHARACTERISTICS**

1. Age ______Years

2. Gender: 1. Male 2. Female

3. Religion of respondent ____________________

4. Ethnicity of respondent ___________________

5. Permanent place of residence:

1. Urban

2. Rural

6. What is your level of education?

a) Lacking formal education

b) Primary level

c) Secondary level

d) College/degree level

7. What is your employment status?

a) Formal employment

b) Self-employment

c) Peasant

d) No employment

8. What kind of marriage are you currently in?

a) Living with partner (married, cohabiting)

b) Living without partner (Single, divorced, widowed)

9. What is your approximately income per month?

a) Less than 300,000/=

b) More than 300,000/=

c) No official income

**PART II: ORGAN DONATION KNOWLEDGE QUESTIONS**

10. Have you ever heard of kidney donation?

a. yes b. no

If yes,

11. What is the source of information? Circle all applied

a. television

b. internet

c. social networking

d. health care workers

e. friends

f. family member

12. What does kidney donation mean to you?

a. Transfer of a kidney from a dead body to a patient in need

b. Transfer of a kidney from a living donor to a patient in need

c. All of the above

d. Don’t know

13. Apart from kidney, what other organs/tissues can be donated?

a. Heart g. Cornea of the Eyes m. Don’t know

b. Liver h. Skin

c. Lungs i. Bone marrow.

d. Pancreas j. Bone

e. Intestine k. All of above

f. Blood l. None of the above

14. At what age can an individual donate a kidney?

a) At any age

b) 18 years and above

c) Don’t know

15. Does your religion allow organ donation?

a. Yes b. No c. don’t know

16. Do you know anyone who has donated an organ?

a. Family member b. Friend c. Colleague d. No one

17. Do you know that you can donate one of your two kidneys during your life, to another person?

a. Yes b. No

18. Do you know that donating a kidney is safe?

a. Yes b. No c. Maybe d. don’t know

19. Do you know that you can donate all your two kidneys after death?

a. Yes b. No

20. Diabetes and Hypertension are the most common causes for people to require kidney transplant?

a. Yes b. No

21. Is it feasible for someone who is brain dead to recover from their injuries?

a. Yes b. No

22. Have you ever had a patient with chronic disease?

a. Yes b. No c. Don’t know

23. Have you ever cared a relative with kidney disease?

a. Yes b. No

**PART III: ATTITUDES.**

The following statement describes how you personally feel about organ. Place a corresponding response in front of the statement. Likert questions with responses 1) Strong agree 2) Agree 3) neither agree nor disagree 4) Disagree and 5) Strongly agree.

| Statement | 1 | 2 | 3 | 4 | 5 |
| --- | --- | --- | --- | --- | --- |
| 24. Organ donation is a good thing and should be promoted |  |  |  |  |  |
| 25. Donating an organ could save somebody’s life |  |  |  |  |  |
| 26. I think my donation whether living or after death is going to impact my life after death in a good way |  |  |  |  |  |
| 27. Organ donation is an act which will be rewarded by God |  |  |  |  |  |
| 28. My family will not allow donation of my organs |  |  |  |  |  |
| 29. You are worried that organ donation might leave you weak and disabled |  |  |  |  |  |
| 30. I don’t trust the health care system and it is better to go abroad for organ donation and organ transplantation |  |  |  |  |  |
| 31. You are not healthy to donate |  |  |  |  |  |
| 32. Your age is not fit for donating your organ. |  |  |  |  |  |
| 33. Operation procedure for procuring organs is discouraging |  |  |  |  |  |
| 34. Organ retrieval process after death may cause body disfigurement |  |  |  |  |  |
| 35. In case of an emergency, doctors will not provide enough care if the patient is a known organ donor |  |  |  |  |  |
| 36. I believe in the burial of intact body |  |  |  |  |  |

**PART VI: WILLINGNESS TO DONATE ORGAN**

37. Are you willing to donate your kidney?

a. Yes b. No

If yes, answer question number 38 and 39

38. Which type of donation will you prefer? Tick all applied

a. Living donation b. Deceased donation

39. Are you willing to consent for organ donation of a family member after their death?

a. Yes b. No
