# Supplementary material for: Willingness toward kidney donation among patients’ relatives at Muhimbili National Hospital, Dar es Salaam, Tanzania: A cross-sectional study
Source: PLoS One. 2026 Jul 10;21(7):e0351952. doi: 10.1371/journal.pone.0351952 (PMC13353935; doi:10.1371/journal.pone.0351952)
Supplement: S10 File — (DOCX) [file pone.0351952.s010.docx]

***PLOS ONE* Clinical Studies Checklist**

***PLOS ONE* manuscript number: PONE-D-23-00901**

| **Complete the following if your study involved human participants or human subjects’ data. These questions should be addressed for prospective and retrospective studies.** | | |
| --- | --- | --- |
| 1. | Did you obtain ethics approval for this study?   - If yes, please upload (file type “Other”) the original approval document you received from your ethics committee. If the original document is in another language, please also provide an English translation. - *Yes, ethics approval letters have been received to conduct the study and the document has been uploaded.* - *A statement that describes the ethics approval has been mentioned in the* ***“Ethics and dissemination”*** *section of the abstract and in the* ***“Ethics”*** *of the Methods sections. Both the ethics approvals were written and provided in the English language.* - If you did not obtain ethical approval, please explain why this was not required.  \|  \| \| --- \| |  |
| 2. | If your study involved human participants, please report in the Methods section when participants were recruited to the study. *The study involves human participants and the recruitment plan has been mentioned in the* subheading ***“Recruitment”*** in the method section. |  |
| 3. | If you are reporting a study of medical records or archived samples, please report in the Methods section the date range in which human subjects’ data/samples were collected and the date(s) when you conducted this study.  **N/A** |  |
| 4. | Please specify in the Methods section whether authors had access to information that could identify individual participants during or after data collection.  *All information related to study participants will remain confidential and will be identifiable by codes known only to the researcher. This information has been specified in the “****Authors access to study participant information and confidentiality****” subheading in the method section.* |  |
| 5. | If you are reporting an observational study – i.e. cohort, case-control, and cross-sectional studies – we recommend that the work is reported as per the requirements of the STROBE guidelines, and that you provide a completed STROBE checklist as a Supporting Information file with your submission.  The STROBE checklist was developed to improve the reporting of observational human subjects research, and is available here: <http://strobe-statement.org/fileadmin/Strobe/uploads/checklists/STROBE_checklist_v4_combined_PlosMedicine.docx>.  *This study will adhere to the Transparent Reporting of Evaluations with Nonrandomized Designs (TREND) guidelines.* |  |
| 6. | Please ensure that the author list and Corresponding Author entered in Editorial Manager match the author list and Corresponding Author in your manuscript file.  We assure you that the author list and the corresponding authors entered in the Editorial Manager matches with the authors list and corresponding author in the manuscript as indicated below.  Maua Nyagawa^1*^, Baraka Morris^2^, Francis Furia^3^  Affiliation:  ^1^Department of Clinical Nursing (Nephrology Nursing), School of Nursing, Muhimbili University of Health and Allied Sciences (MUHAS), Dar es Salaam, Tanzania.  ^2^Department of Nursing Management, School of Nursing, Muhimbili University of Health and Allied Sciences (MUHAS), Dar es Salaam, Tanzania.  ^3^Department of Pediatrics and Child Health, School of Medicine, Muhimbili University of Health and Allied Sciences (MUHAS), Dar es Salaam, Tanzania.  .  **Completed** |  |
